# Relationship of serum total cholesterol and triglyceride with risk of mortality in maintenance hemodialysis patients: a multicenter prospective cohort study

**DOI:** 10.1080/0886022X.2024.2334912

**Published:** 2024-04-11

**Authors:** Yan Huang, Qiuxia Zhong, Junzhi Chen, Xianhui Qin, Yaya Yang, Yanhuan He, Zizhen Lin, Yumin Li, Shenglin Yang, Yongxin Lu, Yanhong Zhao, Yaozhong Kong, Qijun Wan, Qi Wang, Sheng Huang, Yan Liu, Aiqun Liu, Fanna Liu, Fanfan Hou, Min Liang

**Affiliations:** a Division of Nephrology, Nanfang Hospital, Southern Medical University, Guangzhou, China; b National Clinical Research Center for Kidney Disease, Guangzhou, China; c State Key Laboratory of Organ Failure Research, Guangzhou, China; d Guangdong Provincial Institute of Nephrology, Guangzhou, China; e Guangdong Provincial Key Laboratory of Renal Failure Research, Guangzhou, China; f People’s Hospital of Yuxi City, Yuxi, China; g The First People’s Hospital of Foshan, Foshan, China; h The Second People’s Hospital of Shenzhen, Shenzhen, China; i Huadu District People’s Hospital of Guangzhou, Guangzhou, China; j Nanhai District People’s Hospital of Foshan, Foshan, China; k Guangzhou Red Cross Hospital, Guangzhou, China; l The Third Affiliated Hospital of Southern Medical University, Guangzhou, China; m Guangzhou Overseas Chinese Hospital, Guangzhou, China

**Keywords:** Serum total cholesterol, serum triglyceride, maintenance hemodialysis, mortality, cardiovascular mortality

## Abstract

**Objective:**

The relationship between serum total cholesterol (TC) and triglyceride (TG) levels and mortality in maintenance hemodialysis (MHD) patients remains inconsistent. We aimed to explore the individual and combined association of TC and TG levels with the risk of mortality in Chinese MHD patients.

**Methods:**

1036 MHD patients were enrolled in this multicenter, prospective cohort study. The serum levels of total cholesterol and triglycerides were measured at baseline. The primary outcome was all-cause mortality and secondary outcome was cardiovascular disease (CVD) mortality.

**Results:**

During a median follow-up duration of 4.4 years (IQR= 2.0–7.9 years), 549 (53.0%) patients died, and 297 (28.7%) deaths were attributed to CVD. Compared with patients with TC levels in the first three quartiles (<182.5 mg/dL), a significantly higher risk of all-cause mortality was found in participants with TC in the fourth quartile (hazard ratio [HR], 1.43; 95% confidence interval [CI], 1.17–1.76). However, a significantly lower risk of all-cause mortality was observed in participants with TG in the fourth quartile (≥193.9 mg/dL) (HR, 0.78; 95%CI: 0.63–0.98), compared with participants with TG in the first three quartiles. Similar trends were observed in CVD mortality. When analyzed jointly, patients with lower TC (<182.5 mg/dL) and higher TG (≥193.9 mg/dL) levels had the lowest risk of all-cause mortality and CVD mortality.

**Conclusions: **In MHD patients in southern China, higher TC levels were associated with higher risk of mortality, while higher TG levels were related to lower risk of mortality. Patients with lower TC and higher TG levels had the best survival prognosis.

## Introduction

The number of patients with end stage renal disease (ESRD) requiring maintenance dialysis is climbing high throughout the world [1]. Despite gradual improvements in dialysis treatment, patients undergoing maintenance hemodialysis (MHD) continue to experience an annual mortality rate as high as 20% [2], and cardiovascular disease (CVD) accounts for about half of the total mortality [3]. Therefore, identifying potential modifiable mortality risk factors for CVD among patients on MHD will provide a scientific background for prevention.

Certain dyslipidemia patterns, such as elevated serum total cholesterol (TC) and triglyceride (TG) levels, are well recognized as conventional risk factors for CVD in the general population [4–7], which cause the initiation and progression of atherosclerotic vascular disease. However, higher TC and TG levels appear to have paradoxical effect on the mortality risk of patients with ESRD. Several studies have reported that low TC levels are associated with poor outcome in dialysis patients, while hypercholesterolemia is paradoxical related to better survival [8–11], so-called ‘reverse epidemiology’. Data from the relationships between TG levels and mortality in ESRD patients were also in agreement with the reverse epidemiology [9,12]. In contrast, some other previous studies showed that higher TC level was associated with higher morbidity and mortality in MHD patients, as in the general population [13,14]. And elevated TG level in peritoneal patients was corelated with worse survival rates, according to a observational study [15]. To date, the association between lipid levels and survival of dialysis patients still remains controversial and unclear.

The aforementioned results were inconclusive may be due to the cofounding effects of malnutrition and inflammation [16], limited sample size, both maintenance hemodialysis and peritoneal dialysis treatment, and short duration of follow-up [10]. In addition, most previous observational studies examined cohorts of Caucasian or Black dialysis patients before the twenty first century [17]. And previous research has revealed that the paradoxical relationship between serum lipids and mortality in dialysis patients was also modified by race [12,17]. Hence, their findings may not be generalized to a wider population of patients undergoing MHD. Of note, there is a paucity of studies have considered the joint association of TC and TG with mortality risk in MHD patients. To address this knowledge gap, we aimed to explored the individual and combined association of serum total cholesterol and triglyceride levels with the risk of mortality in Chinese MHD patients and tried to find any potential effect modifiers.

## Patients and methods

### Study design and population

Details of the study design have been previously reported [14,15]. In short, this was a multicenter prospective cohort study designed to evaluate dietary nutrition status and its impact on the prognosis of MHD patients. Participants were enrolled from January 2014 to December 2015 in eight outpatient dialysis centers.

The inclusion criteria were aged more than 18 years, undergone MHD for at least 3 months, and had a normal oral dietary intake (not receiving enteral or parenteral). The exclusion criteria included a history of hyperthyroidism, advanced malignant tumor, acute infection, active autoimmune disease, liver cirrhosis, multiple organ failure, gastrointestinal disease, and cognitive disorder.

The study was approved by the Medical Ethics Committee and all participants provided informed consent.

### Data collection and measurements

Demographic data (age, sex, and dialysis vintage), comorbidities (diabetes mellitus, hypertension, and history of CVD), medical history, smoking status, alcohol consumption, and education level were collected by trained research staff according to standardized procedures at enrollment. Diabetes was defined as a medical history of diabetes or the use of glucose-lowing therapy. Participants with a medical history of hypertension or use antihypertensive drugs were defined as having hypertension. A history of CVD was defined as a history of myocardial infarction, heart failure, stroke, transient ischemic attack, cerebrovascular accident, and peripheral arterial disease. Protein energy waste (PEW) was defined according to the International Society of Renal Nutrition and Metabolism (ISRNM) criteria [18].

Anthropometric measurements, including height, weight, waist circumference, and hip circumference, were obtained during physical examination. All measurements were performed by trained research staff after dialysis when the patient had dry weight. Body mass index (BMI) was calculated as the weight/height squared (kg/m^2^).

Blood samples were collected from all participants prior to the hemodialysis session at baseline. Biochemical parameters, including serum albumin, hemoglobin, triglyceride, total cholesterol, blood urea nitrogen (BUN), calcium, phosphate, total carbon dioxide (TCO2), C-reactive protein (CRP), serum creatinine, serum transferrin, were evaluated using an autoanalyzer with standard procedures at each local dialysis center. Kt/V (single pool) was calculated using the urea kinetic modeling (UKM) formula:

Kt/V=− ln (R−0.008×t)+[(4−3.5×R)×UF/W]

where R is the ratio of post-dialysis to pre-dialysis serum urea nitrogen, t is the time of dialysis in hours, UF is the amount of ultrafiltration (in liters), and W is the post-dialysis weight (in kg).

Dietary intake was assessed by trained interviewers using 24-h dietary recall on three nonconsecutive days (including one dialysis day and two non-dialysis days) within one week. The 24-h dietary recalls are relatively quick assessment modalities to obtain the most recent information about food intake and are widely used in epidemiological research [19]. The US Department of Agriculture developed a 24-h recall method that used a five-step, interviewer-administered, computer-based procedure for obtaining dietary recall. Specific introductions of dietary assessment methodologies have been described previously [20,21]. Dietary nutrient intake was calculated using dietary software (version 2.0; Zhending), and nutrient models were based on the Chinese Food Composition Table compiled by the Chinese Center for Disease Control and Prevention in 2009.

### Study outcomes

The primary outcome of this study was all-cause mortality, defined as death from any reason. The secondary outcome was CVD mortality, defined as death from myocardial infarction, cardiac arrest, stroke, heart failure, malignant arrhythmias, sudden cardiac death, and other known vascular causes. All patients were followed up until death, transfer to peritoneal dialysis (PD), kidney transplantation, lost to follow-up or the end of study on 1 August 2023. Survival data including date and cause of death were obtained by death certificates from hospitals, telephone follow-up as well as linking to the national mortality surveillance system from the Chinese Center for Disease Control and Prevention *via* national identification number of the participants [22].

### Statistical analysis

Continuous variables are presented as means ± standard deviations (SDs) or medians and interquartile ranges (IQR). Categorical variables are described as frequencies and percentages. Differences in baseline characteristics according to TC quartiles were compared using the *χ*^2^ test for categorical, and ANOVA tests or Kruskal–Wallis tests for continuous variables as appropriate.

Cox proportional hazards models were used to estimate the hazard ratio (HR) and 95% confidence interval (CI) for the risk of all-cause and CVD mortality associated with TC and TG, respectively, with and without adjustment for sex, dialysis center, age, history of CVD, diabetes, hypertension, smoking status, alcohol consumption, lipid-lowering agent use, dialysis vintage, Kt/V ratio, BMI, waist:hip ratio, serum albumin, serum calcium, hemoglobin, C-reactive protein (CRP, log_10_ transformed), dietary energy intake (DEI, kcal/kg/d, normalized by actual body weight), dietary cholesterol intake and serum triglyceride or serum total cholesterol. The Kaplan-Meier method was used to calculate survival curves, and the log-rank test was used to evaluate survival differences between the TC and TG quartiles, respectively.

In a stratified analysis, possible modifications of the association of TC and TG with all-cause mortality and CVD mortality were assessed for the variables, including age (﹤60 *v*. ≥60 years), sex, BMI (﹤23 *v*. ≥23 kg/m^2^), diabetes (yes *v*. no), history of CVD (yes *v*. no), protein energy waste (PEW) (yes *v*. no), lipid-lowering agent use (yes *v*. no), serum albumin (ALB) (﹤3.8 *v*. ≥3.8 g/dL), and CRP (﹤5 *v*. ≥5 mg/L) levels at baseline.

In sensitivity analyses, to minimize possible effects of the lipid-lowering agents, all hazard ratios were recalculated after excluding the patients who have already taken lipid-lowering agents. Additionally, we have further recalculated the hazard ratios after excluding outliers of total cholesterol and triglyceride levels with more than 3 standard deviations from the mean [23].

Statistical *p* value was set at a two-tailed *p*-value of 0.05. All data analyses were performed using Empower (R) (www.empowerstats.com; X&Y Solution, Inc., Boston, MA), R software, version 3.5.2 (http://www.r-project.org), and GraphPad Prism version 8 (GraphPad Software Inc.).

## Results

### Baseline characteristics of the participants

As illustrated in the flow chart, a total of 1302 adult MHD patients were enrolled in the cohort. After excluding participants who did not qualify for enrollment or missing critical variables, 1036 patients were included in the final analysis (shown in Supplementary Figure 1). The baseline characteristics of patients categorized according to total cholesterol quartiles are shown in Table 1. The mean age of the entire population was 54.1 ± 15.1 years and 57.9% were men. The average levels of TC and TG were 159.9 and 164.4 mg/dL, respectively. Baseline TC levels were positively associated with age, prevalence of current alcohol drinking and diabetes, BMI, WHR, triglyceride, calcium, hemoglobin, and were inversely associated with male gender, prevalence of current smoking (shown in Table 1). While baseline TG levels were positively with age, prevalence of diabetes mellitus and lipid-lowering agent use, BMI, WHR, TC, hemoglobin, dietary protein and dietary energy intake, and were inversely associated with male gender, prevalence of hypertension (shown in Supplementary Table 1).

**Table 1.** The basic characteristics of study participants.

Variables	Total	Total cholesterol, mg/dL				*p*-Value
		Q1 (<131.1)	Q2 (131.5–154.7)	Q3 (155.1–182.5)	Q4 (≥182.9)	
*N*	1036	256	256	260	264	
Demographics						
Age, years	54.1 ± 15.1	50.6 ± 16.2	52.4 ± 14.9	54.8 ± 14.8	58.4 ± 13.4	<0.001
Male, *n*(%)	600 (57.9)	177 (69.1)	168 (65.6)	138 (53.1)	117 (44.3)	<0.001
Current smoking, *n*(%)	150 (14.5)	53 (20.7)	43 (16.8)	26 (10.0)	28 (10.6)	0.008
Current alcohol drinking, *n*(%)	38 (3.7)	8 (3.1)	8 (3.1)	10 (3.8)	12 (4.5)	0.005
Diabetes, *n*(%)	277 (26.7)	68 (26.6)	56 (21.9)	58 (22.3)	95 (36.0)	<0.001
Hypertension, *n*(%)	891 (86.0)	219 (85.5)	218 (85.2)	225 (86.5)	229 (86.7)	0.945
History of CVD, *n*(%)	200 (19.3)	54 (21.1)	39 (15.2)	47 (18.1)	60 (22.7)	0.140
Lipid-lowering agent use, *n*(%)						0.678
Statins	61 (5.9)	11 (4.3)	15 (5.9)	16 (6.2)	19 (7.2)	
Fenofibrate	4 (0.4)	1 (0.4)	0 (0.0)	1 (0.4)	2 (0.8)	
Dialysis vintage, months†	24.4 (12.4-50.5)	21.6 (11.8-50.5)	24.3 (13.4-45.5)	29.6 (12.5-55.3)	23.6 (11.3-49.4)	0.200
Physical examination						
BMI, kg/m^2^	21.2 ± 3.4	20.8 ± 3.1	21.1 ± 3.4	21.0 ± 3.1	22.1 ± 3.7	<0.001
WHR	0.90 ± 0.07	0.89 ± 0.08	0.90 ± 0.06	0.90 ± 0.07	0.92 ± 0.07	<0.001
Biochemistry						
Albumin, g/dL	3.81 ± 0.38	3.81 ± 0.38	3.83 ± 0.36	3.80 ± 0.42	3.79 ± 0.37	0.650
Total cholesterol, mg/dL	159.9 ± 43.4	111.3 ± 17.5	143.1 ± 6.7	167.3 ± 7.7	215.9 ± 35.2	<0.001
Triglyceride, mg/dL	164.4 ± 121.8	123.4 ± 80.0	140.2 ± 81.8	156.4 ± 92.8	235.7 ± 172.8	<0.001
Calcium, mg/dL	8.7 ± 1.0	8.5 ± 1.0	8.6 ± 1.0	8.8 ± 1.1	8.9 ± 1.1	<0.001
Phosphate, mg/dL	6.6 ± 2.0	6.4 ± 2.0	6.7 ± 2.0	6.6 ± 2.0	6.8 ± 1.9	0.265
Hemoglobin, g/dL	10.7 ± 2.1	10.5 ± 2.2	10.9 ± 2.0	10.6 ± 2.1	10.9 ± 2.0	0.045
CRP, mg/L†	2.7 (1.0-7.3)	2.6 (0.9-7.0)	2.2 (0.9-5.6)	2.4 (0.8-8.0)	3.6 (1.3-9.5)	0.384
Kt/V ratio†	1.30 (1.10-1.50)	1.2 (1.1-1.5)	1.3 (1.1-1.5)	1.3 (1.1-1.5)	1.3 (1.1-1.5)	0.611
Dietary parameters						
DPI, g/kg/d	1.1 ± 0.4	1.1 ± 0.3	1.1 ± 0.3	1.1 ± 0.4	1.0 ± 0.4	0.119
DEI, kcal/kg/d	29.1 ± 8.3	29.4 ± 8.5	28.9 ± 8.3	29.7 ± 8.6	28.4 ± 7.9	0.303
Dietary cholesterol, mg/d	244.4 ± 175.0	231.4 ± 142.7	227.7 ± 152.9	257.8 ± 161.6	260.1 ± 227.1	0.060

Data are expressed as mean ± SD; †data are expressed as median with interquartile range (Q1–Q3).

*Abbreviations*: CVD: cardiovascular diseases; BMI: body mass index; WHR: waist: hip ratio; CRP: C-reactive protein; DPI: dietary protein intake; DEI: dietary energy intake.

### Association between baseline lipids and survival in MHD patients

During a median follow-up duration of 4.4 years (IQR= 2.0–7.9 years), 119 (11.5%) patients underwent kidney transplantation, 6 (0.6%) patients were transferred to peritoneal dialysis, 58 (5.6%) patients were lost to follow-up and 4 (0.4%) patients stopped dialysis treatment. These patients were censored at the last date of follow-up. All-cause mortality occurred in 549 (53.0%) patients and 297 (28.7%) deaths were attributed to CVD mortality.

Kaplan-Meier curves of the cumulative events of all-cause mortality for TC quartiles and TG quartiles are shown in Supplementary Figure 2. Participants with TC levels in the fourth quartile had a remarkably higher risk of mortality (*p* = 0.00032). However, there was no significant difference in the risk for all-cause mortality among patients in the quartiles of TG levels (*p* = 0.96).

**Figure 1.**
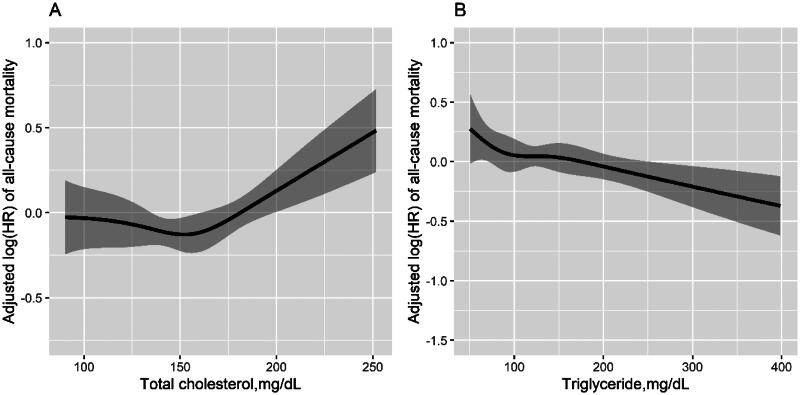
Relationship of serum total cholesterol levels (A) and triglyceride levels (B) with the risk of all-cause mortality*. *Adjusted for age, sex, dialysis center, diabetes, hypertension and history of CVD, smoking, alcohol drinking, lipid-lowering agent use, dialysis vintage, Kt/V ratio, BMI, waist:hip ratio, albumin, serum calcium, hemoglobin, CRP (log_10_ transformed), dietary energy intake, dietary cholesterol intake and serum triglyceride or serum total cholesterol.

The associations between TC and TG levels and all-cause mortality are shown in Figure 1. The multicollinearity test shows that included multiple variables in the models are not redundant (shown in Supplementary Table 2). Overall, in the fully adjusted model (Table 2), when TC was assessed as quartiles, a significantly higher risk for all-cause mortality was found in participants in the fourth quartile (≥182.9 mg/dL: HR, 1.30, 95% CI: 1.00–1.68), compared with those with TC in the first quartile (<131.1 mg/dL). Since the HRs in the first three quartiles were similar, we combined the patients in the first three quartiles into one group. Consistently, compared with those with TC in the first three quartile (<182.5 mg/dL), a remarkably increased risk for all-cause mortality was observed in participants in the fourth quartile (≥182.9 mg/dL: HR, 1.43, 95% CI: 1.17–1.76). Similar results were observed for CVD mortality (Table 2).

**Table 2.** The association between total cholesterol levels and the risk of mortality.

Total cholesterol, mg/dL	No. events (%)	Model I^a^		Model II^b^	
		HR (95%CI)	*p-*Value	HR (95%CI)	*p-*Value
All-cause mortality					
Quartiles					
Q1 (<131.1)	127 (49.6)	1.0 (Ref)	–	1.0 (Ref)	–
Q2 (131.5–154.7)	117 (45.7)	0.81 (0.63, 1.04)	0.100	0.82 (0.63, 1.06)	0.125
Q3 (155.1–182.5)	134 (51.5)	0.94 (0.73, 1.21)	0.630	0.92 (0.71, 1.18)	0.517
Q4 (≥182.9)	171 (64.8)	1.11 (0.88, 1.42)	0.380	1.30 (1.00, 1.68)	0.048
Categories					
Q1–Q3 (<182.5)	378 (49.0)	1.0 (Ref)	–	1.0 (Ref)	–
Q4 (≥182.9)	171 (64.8)	1.22 (1.01, 1.47)	0.040	1.43 (1.17, 1.76)	0.001
CVD mortality					
Quartiles					
Q1 (<131.1)	67 (26.2)	1.0 (Ref)	–	1.0 (Ref)	–
Q2 (131.5–154.7)	64 (25.0)	0.86 (0.61, 1.22)	0.388	0.89 (0.63, 1.26)	0.515
Q3 (155.1–182.5)	75 (28.8)	0.99 (0.71, 1.38)	0.942	0.97 (0.69, 1.37)	0.879
Q4 (≥182.9)	91 (34.5)	1.05 (0.76, 1.47)	0.754	1.30 (0.91, 1.86)	0.154
Categories					
Q1–Q3 (<182.5)	206 (26.7)	1.0 (Ref)	–	1.0 (Ref)	–
Q4 (≥182.9)	91 (34.5)	1.11 (0.86, 1.44)	0.425	1.36 (1.03, 1.80)	0.031

^a^Adjusted for age, sex, dialysis center, diabetes, hypertension and history of CVD. ^b^Adjust for Model I covariates and smoking, alcohol drinking, lipid-lowering agent use, dialysis vintage, Kt/V ratio, BMI, waist:hip ratio, albumin, triglyceride, serum calcium, hemoglobin, CRP (log_10_ transformed), dietary energy intake and dietary cholesterol intake.

Furthermore, after adjustment for the potential confounding factors, when TG was assessed as quartiles, a significantly lower all-cause mortality was detected in patients with TG levels in the fourth quartile (≥193.9 mg/dL: HR, 0.70, 95% CI: 0.52–0.93), compared to the patients in the first quartile (<87.7 mg/dL). Because of the similar HRs in the first three quartiles, we further combined the patients in the first three quartiles into one group. Accordingly, taking the patients in the first three quartiles as the reference, the same results showed a notably decreased risk for all-cause mortality in patients in the fourth quartile (≥193.9 mg/dL: HR, 0.78; 95% CI: 0.63–0.98). Similar trends were observed for CVD mortality (Table 3).

**Table 3.** The association between triglyceride levels and the risk of mortality.

Triglyceride, mg/dL	No. events (%)	Model I^a^		Model II^b^	
		HR(95%CI)	*p-*Value	HR(95%CI)	*p-*Value
All-cause mortality					
Quartiles					
Q1 (<87.7)	134 (52.3)	1.0 (Ref)	–	1.0 (Ref)	–
Q2 (88.5–131.0)	133 (50.8)	0.87 (0.68, 1.11)	0.253	0.85 (0.66, 1.09)	0.210
Q3 (131.9–193.0)	138 (53.3)	0.88 (0.69, 1.13)	0.318	0.88 (0.68, 1.14)	0.334
Q4 (≥193.9)	144 (55.6)	0.80 (0.63, 1.03)	0.081	0.70 (0.52, 0.93)	0.015
Categories					
Q1–Q3 (<193.0)	405 (52.1)	1.0 (Ref)	–	1.0 (Ref)	–
Q4 (≥193.9)	144 (55.6)	0.88 (0.72, 1.07)	0.212	0.78 (0.63, 0.98)	0.033
CVD mortality					
Quartiles					
Q1 (<87.7)	78 (30.5)	1.0 (Ref)	–	1.0 (Ref)	–
Q2 (88.5–131.0)	76 (29.0)	0.80 (0.58, 1.11)	0.184	0.80 (0.58, 1.12)	0.197
Q3 (131.9–193.0)	72 (27.8)	0.75 (0.54, 1.04)	0.084	0.81 (0.57, 1.16)	0.252
Q4 (≥193.9)	71 (27.4)	0.62 (0.44, 0.86)	0.005	0.60 (0.41, 0.90)	0.013
Categories					
Q1–Q3 (<193.0)	226 (29.1)	1.0 (Ref)	–	1.0 (Ref)	–
Q4 (≥193.9)	71 (27.4)	0.74 (0.56, 0.97)	0.031	0.72 (0.53, 0.98)	0.036

^a^Adjusted for age, sex, dialysis center, diabetes, hypertension and history of CVD. ^b^Adjust for Model I covariates and smoking, alcohol drinking, lipid-lowering agent use, dialysis vintage, Kt/V ratio, BMI, waist:hip ratio, albumin, serum total cholesterol, serum calcium, hemoglobin, CRP (log_10_ transformed), dietary energy intake and dietary cholesterol intake.

In the sensitivity analysis, to minimize potential effects of the lipid-lowering drugs, we excluded the participants who used lipid-lowering agents at baseline, the Cox proportional regression results demonstrated above were not materially changed (shown in Supplementary Tables 3 and 4). Similar results were observed after excluding the outliers of total cholesterol and triglyceride (shown in Supplementary Tables 5 and 6).

**Figure 2.**
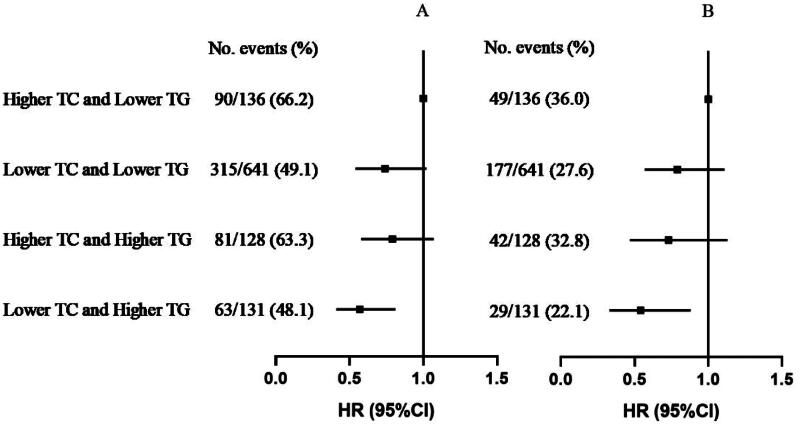
Joint association of TC and TG with all-cause mortality (A) and CVD mortality (B)*. *Adjusted for age, sex, dialysis center, diabetes, hypertension and history of CVD, smoking, alcohol drinking, lipid-lowering agent use, dialysis vintage, Kt/V ratio, BMI, waist:hip ratio, albumin, serum calcium, hemoglobin, CRP (log_10_ transformed), dietary energy intake, dietary cholesterol intake.

Additionally, we analyzed the joint association of TC and TG levels with mortality. Compared to patients with higher TC (quartile 4: ≥182.9 mg/dL) and lower TG (quartile 1–3: <193.9 mg/dL) levels, those with lower TC and lower TG (HR, 0.72; 95%CI, 0.56-0.93) levels, higher TC and higher TG (HR, 0.74; 95%CI, 0.54–1.02) levels and lower TC and higher TG (HR, 0.57; 95%CI, 0.41–0.81) levels had lower risk of all-cause mortality (shown in Figure 2(A)). Similar results were found for CVD mortality (shown in Figure 2(B)).

### Stratified analyses

**Figure 3.**
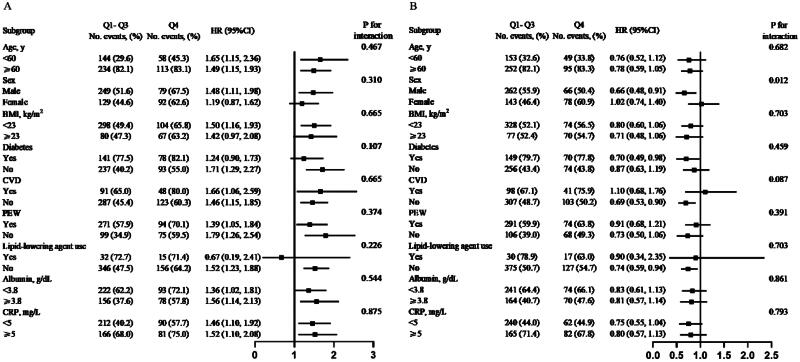
Stratified analysis of the association between serum total cholesterol (A), triglyceride (B) and the risk of all-cause mortality*. *Adjusted for age, sex, dialysis center, diabetes, hypertension and history of CVD, smoking, alcohol drinking, lipid-lowering agent use, dialysis vintage, Kt/V ratio, BMI, waist:hip ratio, albumin, serum calcium, hemoglobin, CRP (log_10_ transformed), dietary energy intake, dietary cholesterol intake and serum triglyceride or serum total cholesterol.

Stratified analyses were performed to assess the association between TC and the risk of all-cause or CVD mortality in various subgroups. None of these variables, including age (<60 *v*. ≥60, years), sex (male *v*. female), BMI (<23*v*. ≥23, kg/m^2^), diabetes (yes *v*. no), history of CVD (yes *v*. no), PEW (yes *v*. no), lipid-lowering agent use (yes *v*. no), ALB (<3.8 *v*. ≥3.8, g/dL), and CRP (<5*v*. ≥5, mg/L), significantly modified the correlation between TC and the risk of all-cause mortality or CVD mortality (all *p* > 0.05, shown in Figure 3(A), Supplementary Figure 3(A)). Similar results were found in the stratified analysis of TG and CVD mortality (all *p* > 0.05), (shown in Supplementary Figure 3(B)). However, we observed a positive correlation between TG levels and all-cause mortality for females, while a negative correlation for males (*p* = 0.012 for interaction, shown in Figure 3(B)).

## Discussion

In this multicenter, prospective cohort study conducted in Chinese patients with MHD, we observed that high TC levels were associated with a significantly increased risk of all-cause mortality and CVD mortality; while high TG levels were inversely associated with decreased risk of all-cause and CVD mortalities. Moreover, when TC and TG were considered together, the patients with lower TC (quartile 1-3: <182.5 mg/dL) and higher TG (quartile 4: ≥193.9 mg/dL) levels had the lowest risk of all-cause and CVD mortality.

Some studies have demonstrated that high TC levels contribute to a greater risk of cardiovascular disease or total mortality in dialysis patients. The study by Lin et al. from Taiwan was a nationwide cohort study covering 90,795 MHD patients and showed that TC >250 mg/dL significantly increased the risk of mortality (HR:1.27; 95%CI: 1.17–1.37) in patients without myocardial infarction or coronary artery disease [13]. And data from the Taiwan Renal Registry Data System also indicated that TC >285 mg/dL was associated with an increased risk of mortality in peritoneal dialysis patients [24]. Consistently, we found that patients with TC in the fourth quartile (≥182.9 mg/dL) had a significantly higher risk of all-cause and CVD mortality. However, multiple observational studies have reported an inconsistent or paradoxical correlation between TC and mortality in patients undergoing MHD [14,25]. Some investigators have proposed several possible explanations for the inverse relationship between TC levels and outcome in dialysis patients. One of the convincing theory is that this ‘reverse epidemiology’ phenomenon is due to the cholesterol-lowering effect of inflammation/malnutrition, rather than the protective effect of high TC levels [16]. Another powerful explanation is the ‘time discrepancy of competitive risk factors’. It has been suggested that hypercholesterolemia appears beneficial in the short term, while it becomes detrimental over a long period. Hence, dialysis patients die because of disorders associated with inflammation and/or malnutrition in a shorter period of time, so that we can found an relationship between low TC levels and mortality [26,27]. The current study has a relatively long follow-up period (median follow-up time: 4.4 years, IQR= 2.0–7.9 years), therefore we observed that high TC levels increased the risk of all-cause and CVD mortality in MHD patients. Our findings are also supported by the *post hoc* analysis of the 4D study [28,29]. März W et al. showed that atorvastatin significantly reduced the risk of fatal and nonfatal cardiac events and death from any cause if the low-density lipoprotein cholesterol (LDL-C) before treatment was more than 145 mg/dL [28]. Another study consistently found that atorvastatin reduced all-cause and the risk of all cardiac events in MHD patients with low cholesterol absorption [29].

Previous studies have reported that high TG levels were paradoxically associated with better survival in MHD patients [12,17,30]. A more recent study originating from the Monitoring Dialysis Outcomes (MONDO) study enrolled 37,250 chronic hemodialysis patients followed up to 4 years and revealed that a higher TG level was independently associated with lower all-cause mortality (HR = 0.86, 95% CI: 0.84–0.89), but the association between TG levels and CVD mortality lost its significance after adjusting for nutrition and inflammatory indices [31]. The present study found that the risk of all-cause mortality and CVD mortality was remarkably decreased in patients with serum TG levels in the fourth quartile (≥193.9 mg/dL), and the inverse relationship did not disappear when further adjusted for albumin and CRP levels. Thus far, the underlying mechanisms that account for the reduced mortality associated with higher TG levels in MHD patients are not clear. To our knowledge, TGs play an indispensable role in numerous physiologic process, which are critical to normal healthy. For instant, evidence suggests that triglycerides are lipids synthesized preferentially when energy intake is sufficient, which is used to meet the immediate energy needs of muscles or to store fatty acids for future energy needs. Hence, high TG levels are associated with adequate nutrient intake and good overall health in patients [32]. While in a malnutrition/inflammation state, as seen in hemodialysis patients, high TG levels may be part of a process of offsetting the risk of cachexia and wasting [33]. Notably, in the context of uremia, inflammation, and oxidative stress, TG may be altered in a structure or function similar to high-density lipoprotein cholesterol (HDL-C) [34], because serum TG levels do not reflect the qualitative characteristics of TG-carrying lipoproteins [30]. It also reminds us that the composition or nature of a certain lipoprotein may be more essential than its quantity for evaluating its effect on mortality. Another potential explanation for the paradoxical correlation between TG and mortality may be the competitive events of cardiovascular disease unrelated to TG, such as small vessel coronary disease, cardiomyopathies, or left ventricular hypertrophy [35]. Unfortunately, detailed information regarding these diseases are not available in our study, which hinders further analysis.

According to the current study, we found that gender was an effect modifier between TG and the risk of all-cause mortality. Of note, the average age of females in this study was 54.6 ± 15.0 years, which was a period approaching menopause. Substantial data indicated that female’s risk of CVD, which is the leading cause of mortality in MHD patients, increased dramatically after menopause [36]. On the other hand, previous studies reported that the prevalence of metabolic syndrome (MetS) which includes high levels of triglycerides was higher in females than males [37]. And MetS was a major contributor to morbidity or mortality throughout the world, including China [38].

In addition, the current study assessed the combined association of TC and TG with all-cause and CVD mortality in patients undergoing hemodialysis. Consistent with the results of the independent association analysis, patients with lower TC and higher TG levels had the lowest all-cause and CVD mortality risk. To our knowledge, this is the first study to evaluate the joint association of TC and TG levels on mortality risk in Chinese MHD patients. This method may be helpful in identifying certain subgroups of the population who are at high risk of mortality and who may benefit from large-scale screening programs [39].

Our study has several strengths. First, this was a multicenter, prospective, large cohort study with a relatively long follow-up of up to 9 years and the availability of detailed data on baseline characteristics, comorbidities, dietary nutrients intake and laboratory variables. Second, we excluded patients using lipid-lowering drugs in the sensitivity analyses, thus limiting the confounding effect of statins in reducing mortality via pathways other than lipid lowering mechanisms [40,41], and the primary results did not materially changed. Third, we made a comprehensive adjustment for confounding factors affecting serum lipid levels such as alcohol consumption, dietary energy intake and dietary cholesterol intake. However, this study also has some limitations. First, LDL-C and HDL-C are both critical indicators of blood lipids. Unfortunately, the participants did not have LDL-C and HDL-C levels reported and as a result data analysis using these parameters could not be performed. Second, the lipid data was recorded at baseline, only once, and the time-varying alterations of lipid levels were unavailable. But, there are some precedents for using single-baseline data to predict future events [42]. Third, the type of vascular access is a significant factor influencing the mortality of hemodialysis patients, while it is regrettable that the specific information of each vascular access was not recorded. Fourth, data regarding whether patients underwent parathyroidectomy was not available in this study, which also plays an important role in the survival of MHD patients. Fifth, given the observational nature of our study design, the effect of residual confounding cannot be excluded completely. Finally, the majority of participants in the present study were from southern China, which may lack generalization to other populations.

In conclusion, our study suggests that higher TC levels were associated with higher risk of mortality, while higher TG levels were related to lower risk of mortality, patients with lower TC and higher TG levels had the greatest survival outcomes. Therefore, more detailed screening is needed for early intervention in particular southern China MHD patients with dyslipidemia. And these results of the current study may provide new insights into lipid management in southern China patients with MHD.

## Supplementary Material

Supplemental Material
