# Supplementary material for: Relationship of serum total cholesterol and triglyceride with risk of mortality in maintenance hemodialysis patients: a multicenter prospective cohort study
Source: Ren Fail. 2024 Apr 11;46(1):2334912. doi: 10.1080/0886022X.2024.2334912 (PMC11011237; doi:10.1080/0886022X.2024.2334912)
Supplement: Supplemental Material [file IRNF_A_2334912_SM3317.pdf]

Supplementary Figure 1: Research population screening flow chart.

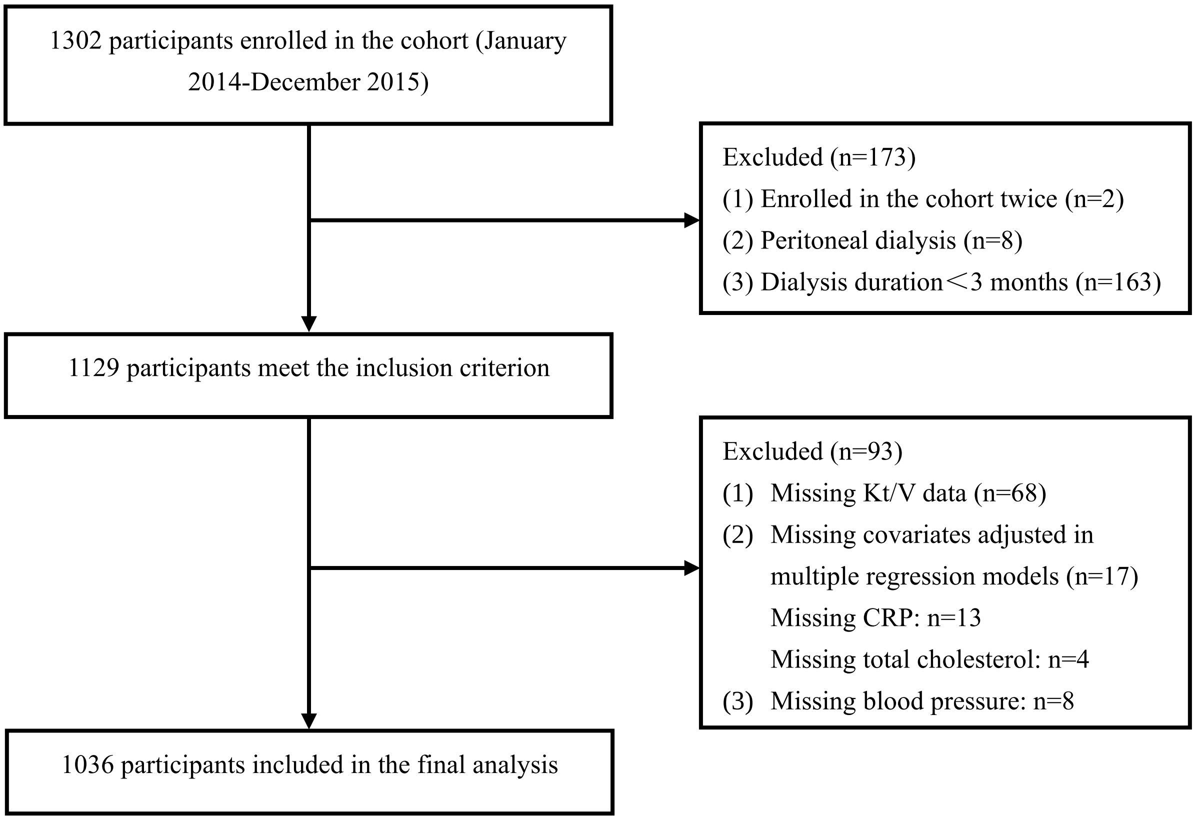

Supplementary Figure 2: Kaplan-Meier analysis of all-cause mortality according to serum total cholesterol quartiles (A) and triglyceride quartiles (B).

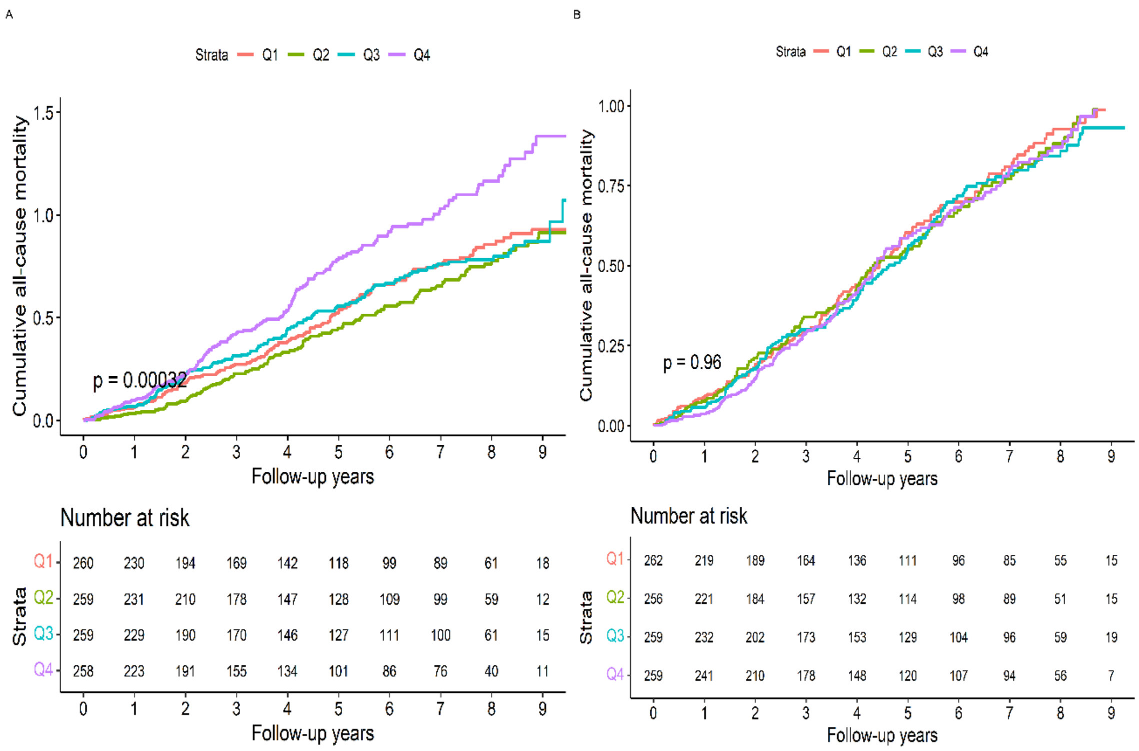

Supplementary Figure 3: Stratified analysis of the association between serum total cholesterol (A), triglyceride (B) and the risk of CVD mortality*.

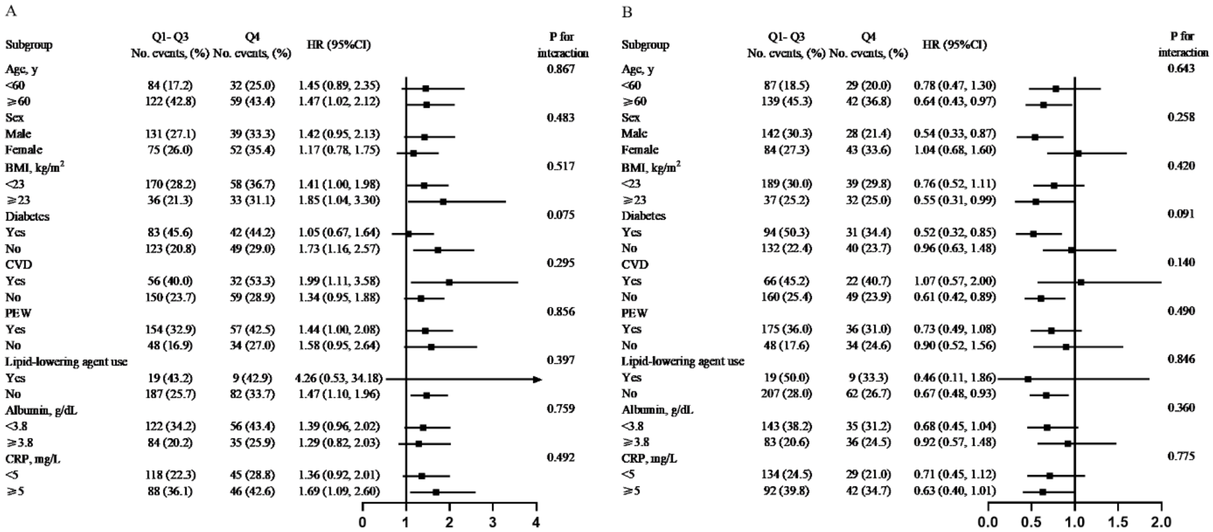

*Adjusted for age, sex, dialysis center, diabetes, hypertension and history of CVD, smoking, alcohol drinking, lipid-lowering agent use, dialysis vintage, Kt/V ratio, BMI, waist:hip ratio, albumin, serum calcium, hemoglobin, CRP (log_{10} transformed), dietary energy intake, dietary cholesterol intake and serum triglyceride or serum total cholesterol.

Supplementary Table 1: The basic characteristics of study participants grouped by triglyceride quartiles.

Variables	Triglyceride, mg/dL				P-value
	Q1(＜87.7)	Q2(88.5-131.0)	Q3(131.9-193.0)	Q4(>=193.9)	
N	256	262	259	259	
**Demographics**					
Age, years	51.6 ± 16.3	54.2 ± 15.7	54.3 ± 14.6	56.1 ± 13.5	0.010
Male, n(%)	162 (63.3)	154 (58.8)	153 (59.1)	131 (50.6)	0.030
Current smoking, n(%)	40 (15.6)	43 (16.4)	33 (12.7)	34 (13.1)	0.837
Current alcohol drinking, n(%)	8 (3.1)	10 (3.8)	10 (3.9)	10 (3.9)	0.352
Diabetes, n(%)	43 (16.8)	67 (25.6)	77 (29.7)	90 (34.7)	<0.001
Hypertension, n(%)	226 (88.3)	237 (90.5)	214 (82.6)	214 (82.6)	0.016
History of CVD, n(%)	46 (18.0)	54 (20.6)	46 (17.8)	54 (20.8)	0.712
Lipid-lowering agent use, n(%)					0.020
Statins	7 (2.7)	15 (5.7)	13 (5.0)	26 (10.0)	
Fenofibrate	1 (0.4)	0 (0.0)	2 (0.8)	1 (0.4)	
Dialysis vintage, months†	23.0 (12.0-47.9)	24.6 (13.5-49.3)	23.5 (11.2-51.6)	27.2 (12.8-51.8)	0.996
**Physical examination**					
BMI, kg/m^2	20.1 ± 2.7	20.3 ± 3.0	21.4 ± 3.4	23.1 ± 3.5	<0.001
WHR	0.88 ± 0.07	0.89 ± 0.07	0.90 ± 0.07	0.94 ± 0.06	<0.001
**Biochemistry**					
Albumin, g/dL	3.78 ± 0.37	3.80 ± 0.36	3.83 ± 0.41	3.83 ± 0.37	0.407
Total cholesterol, mg/dL	141.3 ± 30.9	150.3 ± 35.5	164.2 ± 43.0	183.6 ± 49.9	<0.001
Triglyceride, mg/dL	69.0 ± 13.0	109.5 ± 12.0	159.5 ± 17.7	319.3 ± 150.7	<0.001
Calcium, mg/dL	8.6 ± 0.9	8.7 ± 1.1	8.6 ± 1.1	8.8 ± 1.1	0.118

Phosphate, mg/dL	6.9 ± 2.1	6.4 ± 1.9	6.6 ± 1.9	6.7 ± 2.0	0.051
Hemoglobin, g/dL	10.5 ± 2.1	10.7 ± 2.1	10.8 ± 2.0	11.0 ± 2.0	0.028
CRP, mg/L†	2.4 (0.7-6.2)	1.9 (0.7-6.3)	2.3 (1.1-6.2)	4.6 (1.7-10.4)	0.073
Kt/V ratio†	1.2 (1.1-1.5)	1.3 (1.1-1.5)	1.3 (1.1-1.5)	1.3 (1.1-1.5)	0.066
**Dietary parameters**					
DPI, g/kg/d	1.1 ± 0.4	1.1 ± 0.3	1.1 ± 0.4	1.0 ± 0.3	<0.001
DEI, kcal/kg/d	30.9 ± 8.8	29.5 ± 8.2	28.9 ± 7.8	27.2 ± 8.1	<0.001
Dietary cholesterol, mg/d	248.9 ± 145.8	244.3 ± 158.5	250.8 ± 230.7	233.8 ± 151.8	0.693

Data are expressed as mean ± SD; †: Data are expressed as median with interquartile range (Q1 to Q3).

Abbreviations: CVD: cardiovascular diseases; BMI: body mass index; WHR: waist:hip ratio; CRP: C-reactive protein; DPI: dietary protein intake; DEI: dietary energy intake.

Supplementary Table 2. The variance inflation factors for the included variables

Variables	Variance inflation factor (VIF)
Age, years	1.4
Sex, n(%)	1.2
center, n(%)	1.2
Diabetes, n(%)	1.2
Hypertension, n(%)	1.1
History of CVD, n(%)	1.1
Smoking status, n(%)	1.1
Alcohol drinking status, n(%)	1.2
Lipid-lowering agent use, n(%)	1.1
Dialysis vintage, months	1.1
Kt/V ratio	1.2
Body mass index, kg/m^2	1.7
Waist:hip ratio	1.5
Albumin, g/dL	1.3
Triglyceride, mg/dL	1.2
Calcium, mg/dL	1.1
Hemoglobin, g/dL	1.1
C-reactive protein, mg/L	1.2
Dietary energy intake, kcal/kg/d	1.4
Dietary cholesterol, mg/d	1.1

Supplementary Table 3: The associations of total cholesterol levels with all-cause and cardiovascular mortality after excluding the patients with the lipid-lowering agents used.

Total cholesterol, mg/dL	No. events (%)	Model I [a]		Model II [b]	
		HR(95%CI)	*P*-value	HR(95%CI)	*P*-value
**All-cause mortality**					
Quartiles					
Q1 (<130.7)	118 (48.6)	1.0 (Ref)	-	1.0 (Ref)	-
Q2 (131.5-154.7)	106 (43.8)	0.80 (0.61, 1.04)	0.101	0.81 (0.62, 1.06)	0.132
Q3 (155.1-182.5)	122 (50.2)	0.95 (0.73, 1.23)	0.682	0.90 (0.69, 1.17)	0.414
Q4 (⩾182.9)	156 (64.2)	1.21 (0.94, 1.55)	0.139	1.37 (1.04, 1.78)	0.023
Categories					
Q1-Q3 (<182.5)	346 (47.5)	1.0 (Ref)	-	1.0 (Ref)	-
Q4 (⩾182.9)	156 (64.2)	1.32 (1.08, 1.61)	0.006	1.52 (1.23, 1.88)	<0.001
**CVD mortality**					
Quartiles					
Q1 (<130.7)	63 (25.9)	1.0 (Ref)	-	1.0 (Ref)	-
Q2 (131.5-154.7)	56 (23.1)	0.81 (0.56, 1.17)	0.269	0.85 (0.59, 1.23)	0.393
Q3 (155.1-182.5)	68 (28.0)	0.98 (0.69, 1.39)	0.892	0.93 (0.65, 1.34)	0.708
Q4 (⩾182.9)	82 (33.7)	1.12 (0.80, 1.58)	0.505	1.36 (0.94, 1.97)	0.105
Categories					
Q1-Q3 (<182.5)	187 (25.7)	1.0 (Ref)	-	1.0 (Ref)	-
Q4 (⩾182.9)	82 (33.7)	1.21 (0.92, 1.58)	0.170	1.47 (1.10, 1.96)	0.010

[a] Adjusted for age, sex, dialysis center, diabetes, hypertension and history of CVD. [b] Adjust for Model I covariates and smoking, alcohol drinking, dialysis vintage, Kt/V ratio, BMI, waist:hip ratio, albumin, triglyceride, serum calcium, hemoglobin, CRP ($\log_{10}$ transformed), dietary energy intake and dietary cholesterol intake.

Supplementary Table 4: The associations of triglyceride levels with all-cause and cardiovascular mortality after excluding the patients with the lipid-lowering agents used.

Triglyceride，mg/dL	No. events (%)	Model I [a]		Model II [b]	
		HR(95%CI)	*P*-value	HR(95%CI)	*P*-value
**All-cause mortality**					
Quartiles					
Q1 (<86.8)	123 (50.6)	1.0 (Ref)	-	1.0 (Ref)	-
Q2 (87.7-129.3)	116 (47.9)	0.85 (0.66, 1.10)	0.215	0.81 (0.62, 1.06)	0.125
Q3 (130.2-189.5)	132 (54.5)	0.96 (0.74, 1.23)	0.722	0.92 (0.70, 1.20)	0.521
Q4 (⩾190.4)	131 (53.7)	0.80 (0.62, 1.03)	0.090	0.63 (0.47, 0.85)	0.003
Categories					
Q1-Q3 (<189.5)	371 (51.0)	1.0 (Ref)	-	1.0 (Ref)	-
Q4 (⩾190.4)	131 (53.7)	0.86 (0.70, 1.06)	0.152	0.70 (0.56, 0.89)	0.003
**CVD mortality**					
Quartiles					
Q1 (<86.8)	73 (30.0)	1.0 (Ref)	-	1.0 (Ref)	-
Q2 (87.7-129.3)	64 (26.4)	0.74 (0.53, 1.05)	0.090	0.72 (0.51, 1.03)	0.069
Q3 (130.2-189.5)	68 (28.1)	0.78 (0.55, 1.09)	0.146	0.80 (0.55, 1.15)	0.226
Q4 (⩾190.4)	64 (26.2)	0.60 (0.42, 0.84)	0.004	0.50 (0.33, 0.76)	0.001
Categories					
Q1-Q3 (<189.5)	205 (28.2)	1.0 (Ref)	-	1.0 (Ref)	-
Q4 (⩾190.4)	64 (26.2)	0.72 (0.54, 0.96)	0.025	0.62 (0.45, 0.86)	0.005

[a] Adjusted for age, sex, dialysis center, diabetes, hypertension and history of CVD. [b] Adjust for Model I covariates and smoking, alcohol drinking, dialysis vintage, Kt/V ratio, BMI, waist:hip ratio, albumin, serum total cholesterol, serum calcium, hemoglobin, CRP (log_{10} transformed), dietary energy intake and dietary cholesterol intake.

Supplementary Table 5: The associations of total cholesterol levels with all-cause and cardiovascular mortality after excluding the outliers.

Total cholesterol, mg/dL	No. events (%)	Model I [a]		Model II [b]	
		HR(95%CI)	*P*-value	HR(95%CI)	*P*-value
**All-cause mortality**					
Quartiles					
Q1 (<130.7)	126 (49.8)	1.0 (Ref)	-	1.0 (Ref)	-
Q2 (131.1-154.3)	111 (44.8)	0.79 (0.61, 1.02)	0.068	0.79 (0.61, 1.03)	0.084
Q3 (154.7-181.0)	132 (51.8)	0.93 (0.73, 1.20)	0.599	0.90 (0.70, 1.17)	0.431
Q4 (≥181.7)	163 (64.2)	1.21 (0.95, 1.54)	0.132	1.27 (0.98, 1.65)	0.070
Categories					
Q1-Q3 (<181.0)	369 (48.8)	1.0 (Ref)	-	1.0 (Ref)	-
Q4 (≥181.7)	163 (64.2)	1.33 (1.10, 1.61)	0.003	1.42 (1.16, 1.75)	0.001
**CVD mortality**					
Quartiles					
Q1 (<130.7)	67 (26.5)	1.0 (Ref)	-	1.0 (Ref)	-
Q2 (131.1-154.3)	60 (24.2)	0.82 (0.58, 1.17)	0.282	0.85 (0.59, 1.21)	0.367
Q3 (154.7-181.0)	71 (27.8)	0.94 (0.67, 1.33)	0.732	0.91 (0.64, 1.29)	0.608
Q4 (≥181.7)	93 (36.6)	1.22 (0.87, 1.69)	0.245	1.34 (0.94, 1.92)	0.101
Categories					
Q1-Q3 (<181.0)	198 (26.2)	1.0 (Ref)	-	1.0 (Ref)	-
Q4 (≥181.7)	93 (36.6)	1.32 (1.02, 1.71)	0.034	1.47 (1.12, 1.94)	0.006

[a] Adjusted for age, sex, dialysis center, diabetes, hypertension and history of CVD. [b] Adjust for Model I covariates and smoking, alcohol drinking, lipid-lowering agent use, dialysis vintage, Kt/V ratio, BMI, waist:hip ratio, albumin, triglyceride, serum calcium, hemoglobin, CRP ($\log_{10}$ transformed), dietary energy intake and dietary cholesterol intake.

Supplementary Table 6: The associations of triglyceride levels with all-cause and cardiovascular mortality after excluding the outliers.

Triglyceride，mg/dL	No. events (%)	Model I [a]		Model II [b]	
		HR(95%CI)	*P*-value	HR(95%CI)	*P*-value
**All-cause mortality**					
Quartiles					
Q1 (<86.8)	131 (52.2)	1.0 (Ref)	-	1.0 (Ref)	-
Q2 (87.7-128.4)	128 (50.6)	0.86 (0.67, 1.11)	0.249	0.84 (0.65, 1.08)	0.178
Q3 (129.3-189.5)	138 (54.5)	0.92 (0.72, 1.18)	0.527	0.92 (0.71, 1.20)	0.537
Q4 (⩾190.4)	135 (53.4)	0.83 (0.65, 1.07)	0.153	0.71 (0.53, 0.95)	0.019
Categories					
Q1-Q3 (<189.5)	397 (52.4)	1.0 (Ref)	-	1.0 (Ref)	-
Q4 (⩾190.4)	135 (53.4)	0.90 (0.74, 1.10)	0.310	0.78 (0.62, 0.97)	0.029
**CVD mortality**					
Quartiles					
Q1 (<86.8)	77 (30.7)	1.0 (Ref)	-	1.0 (Ref)	-
Q2 (87.7-128.4)	72 (28.5)	0.78 (0.56, 1.08)	0.133	0.76 (0.54, 1.07)	0.111
Q3 (129.3-189.5)	74 (29.2)	0.80 (0.58, 1.11)	0.184	0.88 (0.62, 1.25)	0.468
Q4 (⩾190.4)	68 (26.9)	0.65 (0.46, 0.91)	0.013	0.61 (0.42, 0.91)	0.014
Categories					
Q1-Q3 (<189.5)	223 (29.5)	1.0 (Ref)	-	1.0 (Ref)	-
Q4 (⩾190.4)	68 (26.9)	0.77 (0.58, 1.01)	0.063	0.71 (0.52, 0.97)	0.032

[a] Adjusted for age, sex, dialysis center, diabetes, hypertension and history of CVD. [b] Adjust for Model I covariates and smoking, alcohol drinking, lipid-lowering agent use, dialysis vintage, Kt/V ratio, BMI, waist:hip ratio, albumin, serum total cholesterol, serum calcium, hemoglobin, CRP ($\log_{10}$ transformed), dietary energy intake and dietary cholesterol intake.
